# The association between community-level economic deprivation and incidences of emergency department visits on account of attempted suicides in Maryland

**DOI:** 10.3389/fpubh.2024.1353283

**Published:** 2024-02-07

**Authors:** Oluwasegun Akinyemi, Temitope Ogundare, Terhas Weldeslase, Tsion Andine, Mojisola Fasokun, Eunice Odusanya, Kakra Hughes, Williams Mallory, Guoyang Luo, Edward Cornwell

**Affiliations:** ^1^Clive O Callender Department of Surgery, Howard University College of Medicine, Washington, DC, United States; ^2^Department of Psychiatry, Boston Medical Center, Boston, MA, United States; ^3^Department of Psychiatry, Boston University Chobanian & Avedisian School of Medicine, Boston, MA, United States; ^4^Department of Surgery, Howard University College of Medicine, Washington, DC, United States; ^5^Department of Epidemiology, School of Public Health, University of Alabama at Birmingham, Birmingham, AL, United States; ^6^Department of Obstetrics and Gynecology, Howard University College of Medicine, Washington, DC, United States

**Keywords:** suicide, distressed community index, United States, suicide prevention, attempted suicide, emergency department

## Abstract

**Background:**

Suicide is a major cause of mortality in the United States, accounting for 14.5 deaths per 100,000 population. Many emergency department (ED) visits in the United States are due to attempted suicides. Suicide attempts predict subsequent completed suicides. Socioeconomic factors, such as community-level socioeconomic deprivation, significantly affect many traditional risk factors for attempted suicides and suicides.

**Aim:**

To determine the association between community-level socioeconomic deprivation and ED visits for attempted suicide in Maryland.

**Methods:**

A retrospective analysis of attempted suicides in the Maryland State Emergency Department Database from January 2018 to December 2020. Community-level socioeconomic deprivation was measured using the Distress Community Index (DCI). Multivariate regression analyses were conducted to identify the association between DCI and attempted suicides/self-harm.

**Results:**

There were 3,564,987 ED visits reported in the study period, with DCI data available for 3,236,568 ED visits; 86.8% were younger than 45 years, 64.8% were females, and 54.6% non-Hispanic Whites. Over the study period, the proportion of ED visits due to attempted suicide was 0.3%. In the multivariate logistic regression, compared to prosperous zones, those in comfortable (OR = 0.80, 95% CI: 0.73–0.88, *p* < 0.01), Mid-Tier (OR = 0.76, 95%CI:0.67–0.86, *p* < 0.01), At-Risk (OR = 0.77; 95%CI: 0.65–0.92, *p* < 0.01) and Distressed zones (OR = 0.53; 95% CI:0.42–0.66, *p* < 0.01) were less likely to visit the ED for attempted suicide.

**Conclusion:**

Prosperous communities had the highest rate of attempted suicides, with the risk of attempted suicide increasing as individuals move from the least prosperous to more prosperous areas.

## 1 Introduction

Suicide accounts for 14.5 deaths per 100,000 population in the United States and is a leading cause of death (1). Suicide attempts, non-fatal self-directed, potentially injurious behaviors with the intent to die significantly predict subsequent completed suicide (2). There are about 130 million emergency department visits in the United States yearly; a significant proportion of this is due to attempted suicides and self-inflicted injuries (3). In 2020, over 1.2 million people over 18 reported a suicide attempt and hundreds of thousands more presented to hospitals with self-harm injuries (4).

Suicide prevention strategies rely on understanding the underlying risk factors. The Center for Disease Control provides a framework for organizing risk and protective factors, the social-ecological model, which can inform prevention strategies (5). The social-ecologic model considers the complex interaction between risk and protective factors divided into four levels: individual, interpersonal, community, and societal. The social-ecological model helps provide additional context for developing effective suicide prevention strategies (6).

Prior work has focused mainly on individual-level risk factors for suicide, such as psychiatric diagnoses; impulsiveness; substance use; previous suicide attempts; self-injurious behaviors; age; gender; the victim of domestic violence; physical, emotional, and sexual abuse; low levels of education; being single; and financial instability (7–17). There is little information on risk factors for suicide in the United States that addresses other levels of the social-ecological model. The available studies were conducted among active duty and veterans (18–20). Given the homogeneity of military units, the results may not apply to non-military populations. The results of these studies demonstrate that social and physical environment increase the risk of suicide.

Community-level factors are bounded to a specific region, setting, or area, such as neighborhoods, schools, or workplaces, and play an essential role in health behaviors and outcomes (5, 6). To develop a comprehensive suicide prevention strategy, a multi-level approach to risk assessment is necessary (21). This study aims to address the gaps in the literature by examining the association between neighborhood socioeconomic status and emergency department visits for suicide attempts in Maryland, United States.

## 2 Materials and methods

We conducted a retrospective analysis of all individuals with a diagnosis of attempted suicides in the Maryland State Emergency Department Database from January 2018 to December 2020. The Maryland State Emergency Department Database contains information on all emergency department visits that do not result in hospitalization and is a state-specific version of the National Inpatient Sample Databases, the largest all-payer inpatient database in the United States, which comprises a 20% stratified random sample of all United States’ hospital discharges. Patients’ information in the database is de-identified.

***Independent variable*** : In this study, the independent variable was the Distress Community Index (DCI). The DCI was created by the Economic Innovation Group and utilizes seven metrics to quantify socioeconomic risk.

These metrics include: (1) the proportion of the population aged 25 years and older without a high school diploma or equivalent; (2) the ratio of housing units that are vacant after adjustment for recreational, seasonal, or occasional use vacancies; (3) the proportion of the population aged 25–54 years who are not working (either unemployed or not in the labor force); (4) the proportion of residents living below the federal poverty rate; (5) the median household income as a percent of a metro area or the state median household income; (6) changes in the number of employees working in the area, and, (7) the number of business establishments in the zip code.

The DCI scores range from 0 (no distress) to 100 (severe distress); they are obtained by ranking ZIP codes on each metric, averaging them, and normalizing data to generate a relative measure of socioeconomic distress. These scores are further stratified into five levels (quintiles) of socioeconomic distress: prosperous, comfortable, mid-tier, at-risk, and distressed.

In this study, patients who did not have complete information to derive the DCI scores and quintiles were excluded from the analysis.

***Primary outcome***: The primary outcome was emergency department visits for attempted suicide coded as a binary variable.

***Covariates***: these included age classified into three groups (<45 years, 45–65 years, and > 65 years), sex, insurance type, race/ethnicity, estimated median household income classified into four quartiles (Quartile I: $1 – $49,999; Quartile II: $50,000 – $64,999; Quartile III: $65,000 – $85,999 and Quartile IV: $86,000+), the presence of psychiatric comorbidities, diagnosis of substance use disorders, preexisting medical conditions such as hypertension, diabetes, and obesity.

### 2.1 Ethical approval

The study was conducted in accordance with the ethical standards of the institutional and/or national research committee and with the 1964 Helsinki declaration and its later amendments or comparable ethical standards. Institutional Review Board approval was waived because the study was carried out on a national database that contained de-identified data and did not require informed consent or direct participation of patients.

### 2.2 Data analysis

Data were analyzed using STATA version 14 (StataCorp College Station, TX). Descriptive statistics such as frequencies and percentages describe patients’ demographics, preexisting comorbidities, and lifestyle behaviors. The chi-square test was used to test the association between categorical variables with DCI and the relationship between DCI quintiles and emergency department visits for attempted suicide. We then conducted multivariate logistic regression analyses to estimate the relationship between DCI and attempted suicide, controlling for covariates. Results are reported using adjusted odd ratios, a 95% confidence interval, and a statistically significant value of *p* < 0.05.

## 3 Results

There were 3,564,987 emergency department visits between January 2018 to December 2020, with DCI information available for 3,236,568. Of these, 8,919 (0.3%) were for attempted suicides. The most common methods (Table 1) were poisoning (55.5%) followed by cutting (38%); 86.8% were younger than 45 years; 64.8% were females; 54.6% were non-Hispanic Whites; 7.7% were uninsured.

**Table 1:** Baseline distribution of study variables.

	Total (*n* = 3,565,018)	Attempted suicides (*n* = 8,919)	No history of attempted suicides (*n* = 3,556,099)	*p* value
Age (Years)				<0.001
< 45	2, 083,492 (58.5%)	7,741 (86.8%)	194 (58.4%)	
45–65	909,988 (25.5%)	983 (11.0%)	909,005 (25.6%)	
> 65	571,267 (16.0%)	194 (2.2%)	571,073 (16.1%)	
Female	1,955,338 (54.9%)	5,773 (64.8%)	1,949,565 (54.8%)	
Race/ethnicity				<0.001
White	1,484,786 (42.0%)	4,812 (54.6%)	1,479,974 (42.0%)	
Black	1,574,247 (44.5%)	2,668 (30.3%)	1,571,579 (44.6%)	
Hispanics	304,336 (8.6%)	764 (8.7%)	303,572 (8.6%)	
Asian/Pacific Islander	64,152 (1.8%)	159 (1.8%)	63,993 (1.8%)	
Native Americans	7,123 (0.2%)	23 (0.3%)	7,100 (0.2%)	
Others	100,320 (2.8%)	386 (4.4%)	99,934 (2.8%)	
Insurance				<0.001
Medicare	705,575 (19.8%)	639 (7.2%)	704,936 (19.8%)	
Medicaid	1,247,408 (35.0%)	3,622 (40.8%)	1,243,786 (35.0%)	
Private	1,114,224 (31.3%)	3,266 (36.8%)	1.110,958 (31.3%)	
Uninsured	322,015 (9.0%)	685 (7.7%)	321,330 (9.0%)	
Others	174,044 (4.9%)	676 (7.6%)	173,368 (4.9%)	
Income				<0.001
Quartile I	563,844 (16.1%)	995 (11.3%)	562,849 (16.0%)	
Quartile II	504,042 (14.3%)	1,126 (12.8%)	502,916 (14.3%)	
Quartile III	1,034,273 (29.4%)	2,397 (27.3%)	1,031,876 (29.4%)	
Quartile IV	1,419,440 (40.3%)	4,258 (48.5%)	1,415,182 (40.3%)	
Distress community index				<0.001
Prosperous	680,878 (21.0%)	2,279 (28.0%)	678,599 (21.0%)	
Comfortable	806,333 (24.9%)	2,172 (26.7%)	804,161 (24.9%)	
Mid-tier	738,459 (22.8%)	1,693 (20.8%)	736,766 (22.8%)	
At risk	394,536 (12.2%)	901 (11.1%)	393,635 (12.2%)	
Distressed	616,391 (19.0%)	1,106 (13.6%)	615,285 (19.1%)	
Comorbidities				
Current smokers	39,683 (1.1%)	123 (1.4%)	39,560 (1.1%)	0.02
Hypertension	777,668 (21.8%)	788 (8.8%)	776,880 (21.9%)	<0.001
Diabetes mellitus	325,047 (9.1%)	279 (3.1%)	324,768 (9.1%)	<0.001
Obesity	120,690 (3.4%)	224 (2.5%)	120,466 (3.4%)	<0.001
Mental health conditions				
Depression	211,007 (5.9%)	4,604 (51.6%)	206,403 (5.8%)	<0.001
Substance abuse	3,261 (0.1%)	9 (0.1%)	3,252 (0.10%)	0.768
Alcohol addiction	47,436 (1.3%)	453 (5.1%)	46,983 (1.3%)	<0.001
Schizophrenia	1,437 (0.0%)	9 (0.1%)	1,428 (0.0%)	0.004
Bipolar	92,744 (2.6%)	1,168 (13.1%)	91,576 (2.6%)	<0.001
Personality disorders	4,351 (12.0%)	271 (3.0%)	4,080 (0.1%)	<0.001
Anxiety disorders	252,219 (7.1%)	2,207 (24.7%)	250,012 (7.0%)	<0.001
Dementia	54,481 (1.5%)	28 (0.3%)	54,453 (1.5%)	<0.001
Mechanism of attempted suicide				<0.001
Poisoning	31,059 (5.6%)	4,463 (55.5%)	26,596 (4.9%)	
Cuts	49,334 (1.5%)	3,060 (38.0%)	46,274 (8.5%)	
Fires	8,457 (1.5%)	123 (1.5%)	8,334 (1.5%)	
Others	462,350 (83.8%)	403 (5.0%)	461,947 (85.1%)	

There was an inverse relationship between DCI and emergency department visits for attempted suicide (*p* < 0.001): Prosperous (28.0%), Comfortable (26.7%), Mid-Tier (20.8%), At-risk (11.1%) and Distressed (13.6%). Among those who presented to the ED for attempted suicide, there was a higher prevalence of depression (51.6% vs. 5.9%, *p* < 0.001); anxiety (24.7% vs. 7.1%, *p* < 0.001); bipolar disorder (13.1% vs. 2.6%); alcohol use disorder (5.1% vs. 1.3%, *p* < 0.001); schizophrenia (0.1% vs. 0.04%, *p* = 0.004); and personality disorders (3.0% vs. 0.1%, *p* < 0.001). There was no difference in other substance use disorders (0.1% vs. 0.1%, *p* = 0.77) and a lower incidence of dementia (0.3% vs. 1.5%, *p* < 0.001).

In the multivariate analysis (Table 2), after controlling for mental health conditions, age, gender, race/ethnicity, comorbid medical conditions, and insurance, we found a statistically significant association between the DCI and emergency department visits for attempted suicides: Comfortable (OR = 0.80, 95%CI = 0.73–0.88, *p* = 0.001), Mid-Tier (OR = 0.76, 95%CI = 0.67–0.86, *p* < 0.001), At-Risk (OR = 0.77, 95%CI = 0.65–0.92, *p* < 0.001), and Distressed (OR = 0.53; 95%CI = 0.42–0.66, *p* < 0.001).

**Table 2:** Logistic regression showing risk factors for ED visits on account of attempted suicides (Maryland SEDD, 2018–2020).

Attempted suicides	Odds ratio	95% CI	Value of *p*
Age			
< 45 Years	Reference		
45-65 Years	0.31	0.27–0.35	<0.001
>65 Years	0.21	0.16–0.28	<0.001
Female (Ref Male)	2.56	2.35–2.78	<0.001
Race/ethnicity			
White	Reference		
Black	1.06	0.96–1.16	0.25
Hispanics	1.44	1.24–1.68	<0.001
Asian/Pacific Islander	1.33	0.99–1.80	0.06
Native Americans	1.55	0.71–3.37	0.27
Others	1.91	1.58–2.33	<0.001
Insurance			
Private	Reference		
Medicare	1.07	0.88–1.30	0.51
Medicaid	0.73	0.67–0.81	0.50
Uninsured	0.61	0.52–0.71	<0.001
Others	0.83	0.69–0.99	0.04
Distress community index			
Prosperous	Reference		
Comfortable	0.83	0.74–0.93	0.001
Mid-tier	0.78	0.69–0.87	<0.001
At risk	0.74	0.64–0.85	<0.001
Distressed	0.56	0.48–0.64	<0.001
Comorbidities			
Smoking	1.11	0.82–1.49	0.51
Hypertension	0.77	0.66–0.89	0.001
Diabetes mellitus	0.90	0.71–1.14	0.38
Obesity	1.12	0.86–1.45	0.40
Mental health condition			
Depression	9.00	8.07–9.97	<0.001
Alcohol abuse	3.45	2.90–4.11	<0.001
Bipolar disorders	5.48	4.74–6.33	<0.001
Personality disorders	12.03	8.01–18.04	<0.001
Anxiety disorders	1.63	1.46–1.83	<0.001
Mechanism of attempted suicide			
Poisoning	Reference		
Cuts	0.53	0.49–0.58	<0.001
Fires	0.13	0.10–0.17	<0.001
Others	0.01	0.01–0.01	<0.001
Constant	0.10	0.09–0.11	<0.001

In addition, the presence of mental health conditions was found to be an independent predictor of attempted suicide with personality disorders (OR = 12.03, 95%CI = 8.01–18.04, *p* < 0.00); depression (OR = 9.00, 95%CI = 8.07–9.97, *p* < 0.001); bipolar disorder (OR = 5.48, 95%CI = 4.74–6.33, *p* < 0.001); alcohol use disorder (OR = 3.45, 95%CI = 2.90–4.11, *p* < 0.001); and anxiety disorder (OR = 1.63, 95%CI = 1.46–1.83, *p* < 0.001) all associated with increased odds of attempted suicide.

## 4 Discussion

This study aimed to determine the association between neighborhood-level socioeconomic status and attempted suicide in Maryland from January 2018 to December 2020. Over the study period, the proportion of ED visits due to attempted suicide was 0.3%, with the largest proportion of emergency department visits for attempted suicide found in people living in prosperous neighborhoods. After controlling for covariates, the odds of attempted suicide were highest among those living in prosperous neighborhoods.

This study had 8,919 emergency department visits for attempted suicide in Maryland. In 2020, there were 1.2 million suicide attempts in the United States and 24,000 in Maryland (22, 23). The lower number of emergency department visits reported in this study may be because not all suicide attempts were severe enough to require an emergency department visit, and not all those who attempt suicide seek help afterward. In addition, the study period coincides with the COVID-19 pandemic, with studies showing a reduction in emergency department visits for suicide attempts (24–26). The study found that younger females had the highest incidence of emergency department visits for attempted suicide, consistent with other studies (8, 27–29). In contrast to Ivey-Stephenson et al. (30), this study found the highest incidence of emergency department visits for attempted suicides among patients with private insurance. This may be due to the self-report nature of their study and not observations of actual suicide attempts, unlike this study.

This study examined community-level determinants of attempted suicide using the DCI, a composite measure that measures socioeconomic risk using seven metrics. We found that as one moved from a distressed community to a more prosperous one, the odds of an emergency department visit for attempted suicide increased. There are very few studies to examine the community-level risk of attempted suicide. Soelling et al., in a study among adolescents, found a high level of community distress in adolescents with high suicide attempts (31). Other studies that have examined community-level factors associated with suicide risk have been done so among veterans and active-military officers and focused on military-related factors (18–20). One study found that units with increased suicide attempts increased an individual’s risk for a suicide attempt (20). This may mean that community-level suicide rates may increase individual-level suicide risk. However, given the homogeneity of military units, this may not apply to non-military populations but is an exciting area for future research. Studies in Germany and Denmark have found inconsistent findings regarding community-level socioeconomic status and suicide risk (32, 33).

Studies examining individual suicide risk factors have found that lower socioeconomic status is associated with increased suicide risk (34–36), while others have not found similar associations (32, 37). A complex interplay between individual and community-level factors may exacerbate or attenuate an individual’s vulnerability and suicide risk (38, 39). It is also possible that individuals in prosperous communities with better insurance are more likely to present to the emergency department after a suicide attempt (38).

This study found an association between mental health conditions such as depression, anxiety, personality disorders, and alcohol dependence and emergency department visits for attempted suicide. This association remained after controlling for other variables in the regression analysis, similar to other studies that showed increased suicide risk in people with mental health conditions (6, 8, 26, 37, 38). Comorbid mental health conditions may mediate the association between community-level socioeconomic risk and emergency department visits for attempted suicide (38). While unlikely, it is possible that among the study population, there is a higher prevalence of mental health conditions in people living in more prosperous neighborhoods. It is also possible that there is an under-recognition and underdiagnosis of mental health conditions in distressed communities, predominantly minority populations, due to stigma, distrust of healthcare, lack of access to mental health services, and utilization of alternative care pathways (32, 39–42). It is also possible that people with comorbid mental health conditions in prosperous neighborhoods who attempt suicide are more likely to present to the emergency department because they already utilize the emergency department for other mental health crises.

### 4.1 Limitations

This study has several limitations. First, the study utilized emergency department visits for attempted suicides which most likely underestimates the number of attempted suicides during the study period. Secondly, the study period coincides with the COVID-19 pandemic, and its impact on suicide attempts and emergency department visits for suicide remains largely unknown (43, 44). It is possible that many people who attempted suicide did not present to the emergency department because of fear of contracting COVID (45, 46). Also, it is possible that COVID-19 had a differential impact on prosperous and distressed communities leading to a modification of individual and community-level suicide risk factors. It is possible that people in prosperous communities experienced more social isolation and loneliness, which elevated their risk for suicide during the study period. Third, not every person who presented to the emergency department for attempted suicide in the study period had data to compute the DCI, which may have led to differential misclassification errors when they were excluded from the study.

Despite these limitations, the study is one of the few studies to examine suicide risk factors at the community level using the CDC’s social-ecological model. The study found a direct relationship between community-level socioeconomic risk and emergency department visits for attempted suicide, with more prosperous neighborhoods at increased risk for emergency department visits for suicide attempts. Further study is needed to determine if this is due to access to health care, better insurance, less stigma, and other systemic factors that make people in more prosperous neighborhoods more likely to utilize health care services or if, indeed, community-level socioeconomic status modifies individual-level suicide risk factors.

## 5 Conclusion

In conclusion, this study demonstrated that over the study period, the highest visits to the emergency department for attempted suicide were in the more prosperous neighborhoods, and suicide incidence rates increased with increasing income. This is an interesting finding highlighting the multifactorial nature of suicide risk factors. This study highlights the need to examine suicide using a public health lens, considering factors other than individual-level risk factors. It is only by doing this that we can begin to understand the complex interplay of suicide risk factors and be able to develop a comprehensive suicide prevention strategy.

## Data availability statement

The data analyzed in this study is subject to the following licenses/restrictions: dataset not publicly available. Requests to access these datasets should be directed to HCUP Central Distributor Team, HCUP-RequestData@ahrq.gov.

## Ethics statement

Ethical approval was not required for the study involving humans in accordance with the local legislation and institutional requirements. Written informed consent to participate in this study was not required from the participants or the participants’ legal guardians/next of kin in accordance with the national legislation and the institutional requirements.

## Author contributions

OA: Conceptualization, Data curation, Formal analysis, Methodology, Writing – original draft. TO: Methodology, Supervision, Writing – original draft, Writing – review & editing. TW: Data curation, Formal analysis, Writing – original draft. TA: Writing – original draft. MF: Writing – review & editing. EO: Writing – review & editing. KH: Supervision, Writing – review & editing. WM: Supervision, Writing – review & editing. GL: Supervision, Writing – review & editing. EC: Supervision, Writing – review & editing.
